# Zileuton, a 5-Lypoxigenase Inhibitor, is Antiparasitic and Prevents Inflammation in the Chronic Stage of Heart Chagas Disease

**DOI:** 10.1021/acsinfecdis.4c00623

**Published:** 2024-11-28

**Authors:** Mayra Fernanda Ricci, Estela Mariana Guimarães Lourenço, Rafaela das Dores Pereira, Ronan Ricardo Sabino Araújo, Fernando Bento Rodrigues Oliveira, Elany Barbosa da Silva, Gabriel Stephani de Oliveira, Mauro Martins Teixeira, Nazareth de Novaes Rocha, Felipe Santiago Chambergo, Danilo Roman-Campos, Jader Santos Cruz, Rafaela Salgado Ferreira, Fabiana Simão Machado

**Affiliations:** † Department of Biochemistry and Immunology, Institute of Biological Sciences, Universidade Federal de Minas Gerais, Belo Horizonte 31270-901, Minas Gerais, Brazil; ‡ Department of Microbiology, Institute of Biomedical Sciences, Universidade de São Paulo, São Paulo 05508-000, Brazil; § Program in Health Sciences: Infectious Diseases and Tropical Medicine/Interdisciplinary Laboratory of Medical Investigation, Faculty of Medicine, Universidade Federal de Minas Gerais, Belo Horizonte 30130-100, Minas Gerais, Brazil; ∥ Department of Physiology and Pharmacology, Biomedical Institute, Universidade Federal Fluminense, Niterói 24020-141, Rio de Janeiro, Brazil; ⊥ School of Arts, Sciences and Humanities, Universidade de São Paulo, São Paulo 03828-000, Brazil; # Department of Biophysics, Universidade Federal de São Paulo, São Paulo 04023-062, Brazil

**Keywords:** Chagas disease, cardiomyopathy, zileuton, epoxide hydrolase, trypanocidal effect, immunoregulation

## Abstract

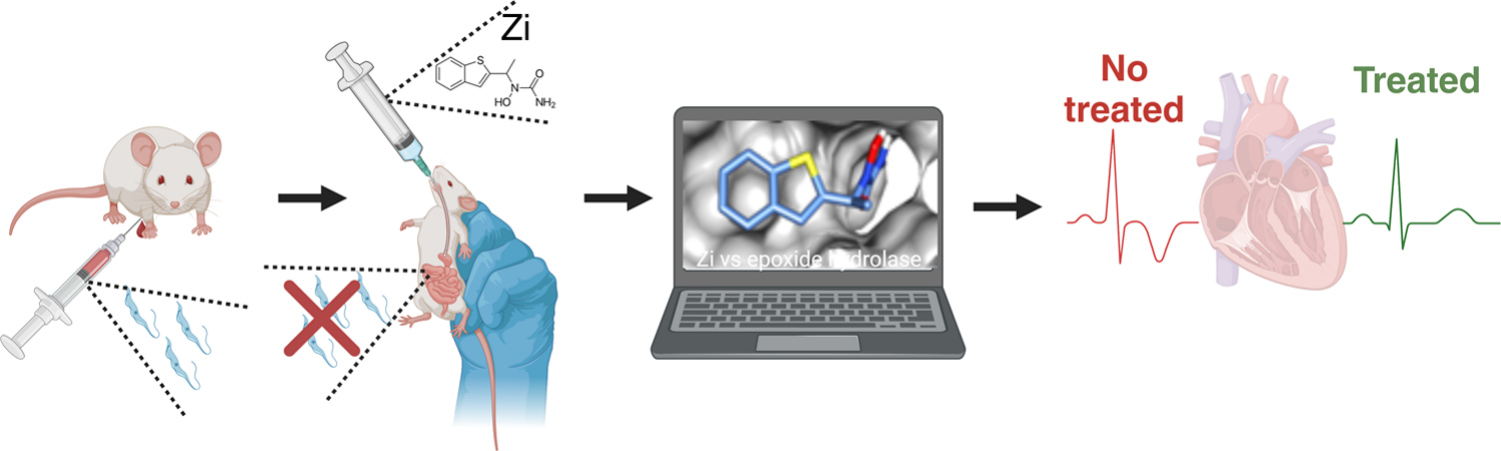

Chronic Chagas cardiomyopathy is associated with an unbalanced immune response and impaired heart function, and available drugs do not prevent its development. Zileuton (Zi), a 5-lypoxigenase inhibitor, affects inflammatory/pro-resolution mediators. Herein, Zi treatment in the early phase of infection reduced parasitemia associated mainly with the direct effect of Zi on the parasite, and the enzyme epoxide hydrolase was the potential molecular target behind the trypanocidal effect. In the intermediate acute phase of infection, Zi reduced the number of innate and adaptive inflammatory cells, increased the level of SOCS2 expression in the heart associated with lower inflammation, and improved cardiac function. Zi treatment initiated in the chronic stage increased the level of SOCS2 expression in the heart, reduced inflammation, and improved cardiac function. Our data suggest that Zi protects against *Trypanosoma cruzi* infection by acting directly on the parasite and reducing heart damage and is a promising option for the treatment of Chagas disease.

Chagas disease is classified as a tropical neglected disease by the World Health Organization and is currently spreading worldwide due to increasing population migration.^1^ It is caused by the protozoan parasite *Trypanosoma cruzi*, which infects virtually all nucleated cells. There are three forms of the parasite: (i) epimastigotes, which are found in the gut of the insect vector; (ii) amastigotes, the replicative form; and (iii) trypomastigotes, which are parasites that infect mammalian host cells, where they can transform into amastigotes and replicate.^2^*T. cruzi* infection induces the production of several inflammatory mediators such as cytokines (e.g., interleukin (IL)-12, tumor necrose factor (TNF), interferon γ (IFN-γ), IL-17, and IL-18), chemokines, reactive oxygen/nitrogen species (ROS/RNS), prostaglandins, leukotrienes, and thromboxanes, among others. During the acute phase of the disease, the presence of these mediators is essential for controlling parasitemia. However, excessive production due to *T. cruzi* infection can lead to significant tissue damage during both the acute and chronic phases of the disease.^3−6^

About 30–40% of infected people develop a symptomatic chronic phase mainly described by the “mega syndromes”: megaesophagus, megacolon, and cardiomyopathy (Chronic Chagasic Cardiomyopathy, CCC). CCC is the most severe and debilitating clinical form of the disease and is mainly caused by strong tissue inflammation, high inflammatory cell infiltration, and fibrosis, resulting in heart failure.^7,8^

Drugs available for treating Chagas disease (benznidazole and nifurtimox) have been used for half a century but present several side effects and compromised efficacy in the chronic phase, which highlights the current necessity of new pharmacological approaches to solving this worldwide health problem.^9^ However, the discovery and development of new drugs are challenging, expensive, and time-consuming. Drug repurposing can reduce these difficulties and has become a central approach in drug development, with successful applications in several diseases, including neglected tropical diseases.^10^

Eicosanoids, such as leukotrienes (LTs) and lipoxins (LXs), are generated through reactions that involve lipoxygenase (LO) and arachidonic acid (AA) metabolism. The enzyme 5-LO plays a pivotal role in the synthesis of LXs and LTs. It is capable of metabolizing AA into 5-hydroperoxy-eicosatetraenoic acid, which is subsequently converted into these mediators.^11,12^ LT participates widely in *T. cruzi* infection by modulating the immune response during the infection.^13^ LXs act as antagonists of LTs’ function and play an anti-inflammatory role in several inflammatory diseases such as tuberculosis, arthritis, toxoplasmosis, intestinal inflammation, and malaria.^14−16^ Additionally, our group demonstrated that LXA_4_ modulates the expression of suppressor of cytokine signaling (SOCS)-2,^17^ and its expression in the heart in a mouse model of *T. cruzi* infection is mainly dependent on the 5-LO signaling pathway.^18^ SOCS2 is a protein with an important role in the regulation of several processes, such as responses to infection, metabolism, and somatic growth.^17,19−22^ Zileuton [(Zi) Zyflo] is a 5-LO inhibitor, considered a safe drug, approved by the Food and Drug Administration (FDA) for asthma prophylaxis and treatment,^23,24^ and studied with promissory results in pathologies such as cancer,^25−27^ severe acne,^28^ murine model of severe acute respiratory syndrome (SARS),^29^ and graft-versus-host disease (GVHD).^30^ This orally administered inhibitor slightly prevents lipid and protein oxidation and is effective against oxidative stress,^31,32^ an important process that also occurs during Chagas disease.

We hypothesized that Zi might have a protective effect in a murine model of CCC. We report the first investigation into the effects of treatment with a selective inhibitor of this enzyme, which has a direct effect on the parasite. Using an *in silico* target-fishing strategy, epoxide hydrolase was identified as a potential trypanocidal molecular target. In the acute stage of infection, Zi treatment reduced the number of macrophages and dendritic cells producing IL-12 and the number of T CD4^+^ and CD8^+^ cells producing IFN-γ and IL-17 in the spleen. Further, Zi treatment during the acute and chronic phases of the disease increased SOCS2 expression in the heart, preserved cardiac architecture, reduced inflammation, and improved cardiac function during the chronic phase. Our results suggest that Zi treatment controls the inflammatory cell profile that may affect the heart, and of great relevance, this drug also demonstrated an antiparasitic effect. These findings indicate that Zi is a potential multitarget drug for the treatment of Chagas heart disease.

## Results

### Treatment with Zi during the Early Acute Phase of *T. cruzi* Infection Reduced the Parasitemia without Changing the Profile of Innate and Adaptive Immune Cells

**Figure 1:**
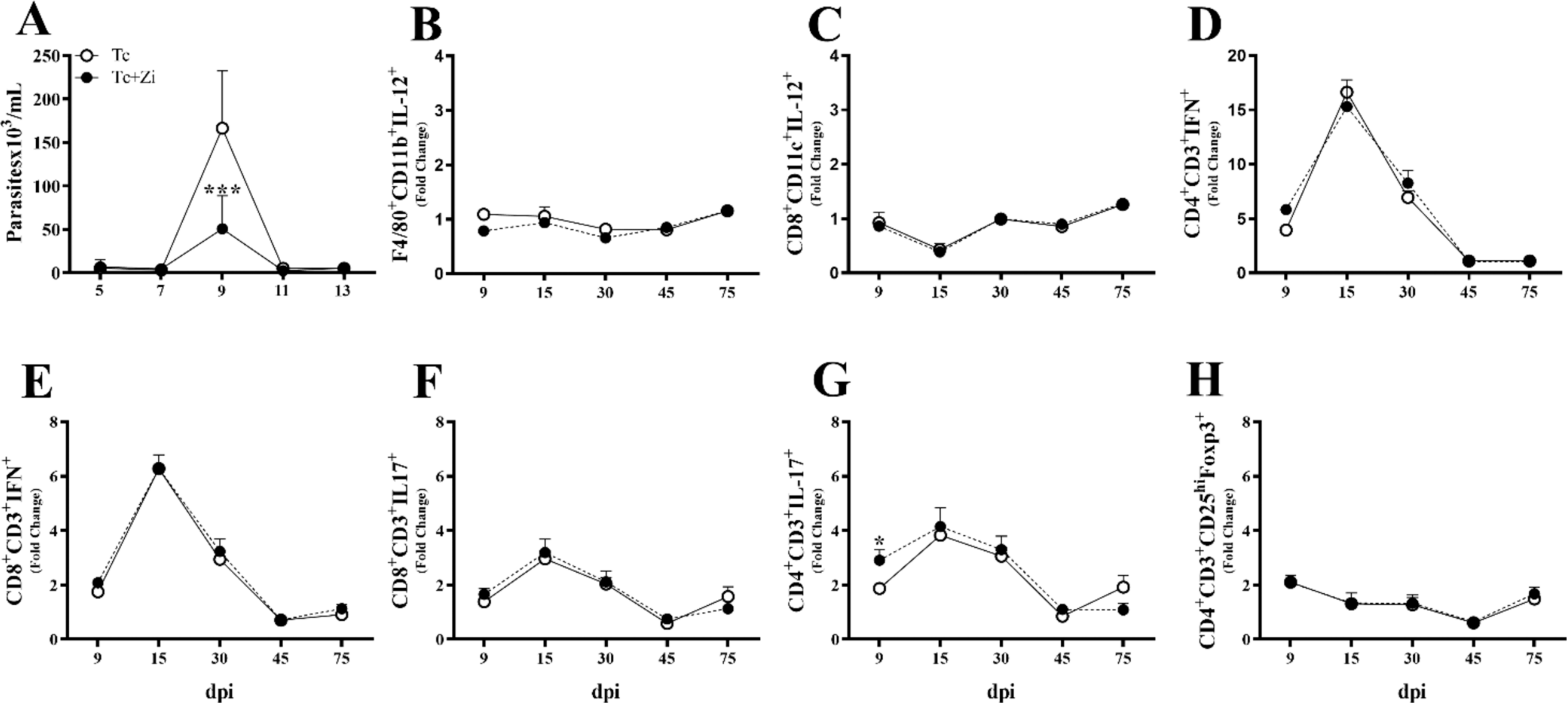
Effect of Zi treatment on parasitemia and the cell profile in the spleen throughout infection. C57BL/6 female mice were infected with 1 × 10^3^ trypomastigotes of *T. cruzi* (strain Y). Mice were treated with Zi from days 3 to 10 after infection. Parasitemia was evaluated at indicated days after infection (dpi) (A); flow cytometry analyses were performed showing the frequency of cells in the spleen at 9, 15, 30, 45, and 75 dpi among groups of macrophages (CD11b^+^ F4/80^+^) producing IL-12 (B); dendritic cells (CD8^+^CD11c^+^) producing IL-12 (**C**), lymphocytes (CD4^+^CD3^+^) producing IFN-γ (D); lymphocytes (CD8^+^CD3^+^) producing IFN-γ (E); lymphocytes (CD8^+^CD3^+^) producing IL-17 (F); lymphocytes (CD4^+^CD3^+^) producing IL-17 (G); T regulatory cells (CD4^+^CD3^+^CD25^hi^Foxp3^+^) (H). * *p* < 0.05; *** *p* < 0.001 (* representation between different groups).

First, we analyzed the effect of Zi treatment on the secondary lymphoid organ, the spleen, at the early/afferent phase (from day 3 to day 10 after infection) of the immune response during *T. cruzi* infection with the Y strain of *T. cruzi*. Zi treatment reduced the peak of parasitemia to 9 days after infection (dpi; Figure 1A). The treatment initiated at the early phase of infection did not change the frequency of macrophages and dendritic cells producing IL-12 and T CD4^+^ and CD8^+^ cells producing IFN-γ and T CD4^+^ regulatory cells in the spleen during all time points evaluated (9, 15, 30, 45, and 75 dpi) (Figure 1B–H), with the exception of the Th17 lymphocytes, which increased at 9 dpi when compared to the untreated infected group (Figure 1G).

### Zi Treatment at the Early Phase of *T. cruzi* Infection Systemically Increased the IFN-γ Levels only after the Peak of Parasitemia and Acted Directly on the Parasite *In Vitro*

**Figure 2:**
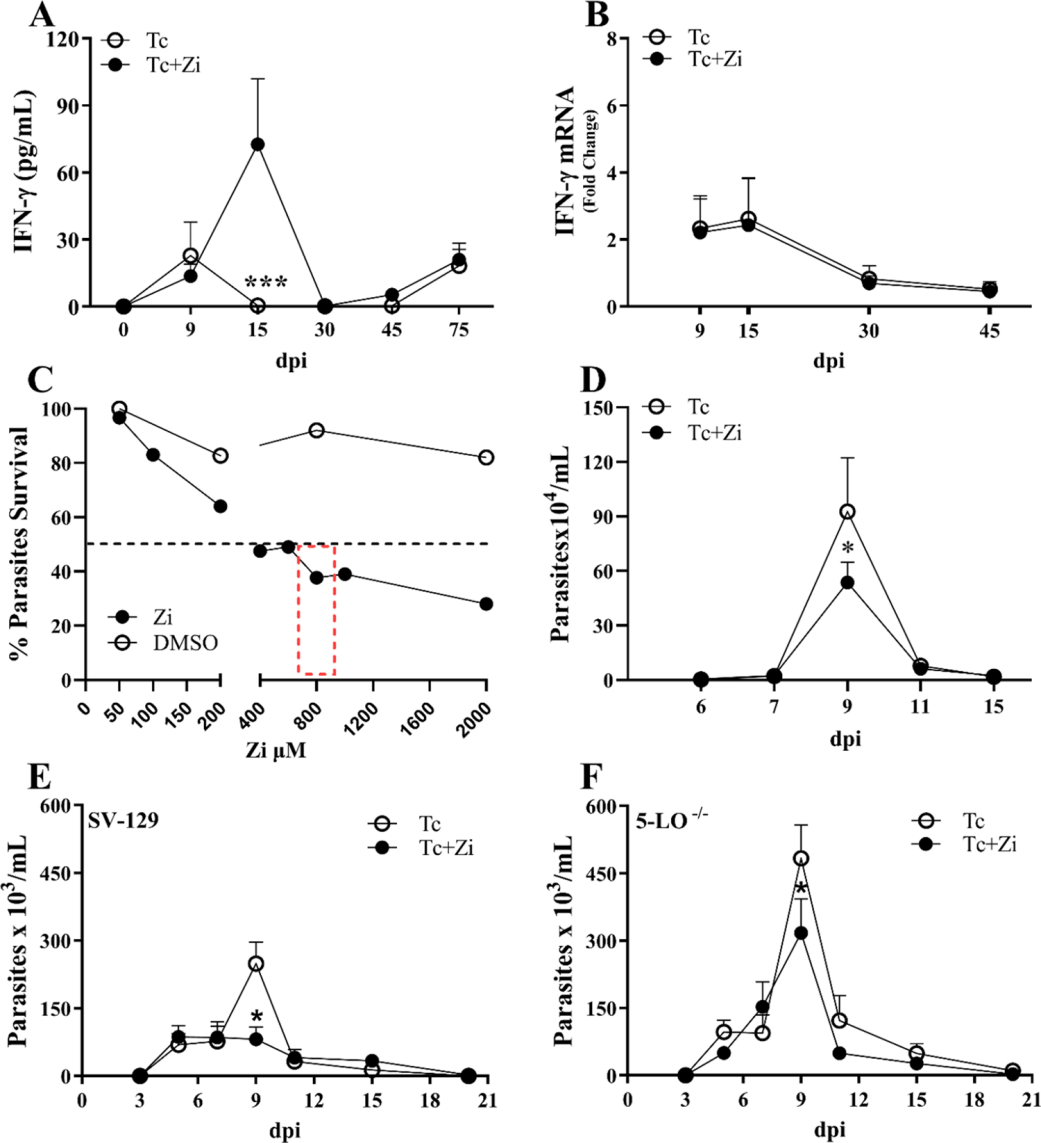
Zi treatment at the early phase of *T. cruzi* infection *in vivo* increased IFN-γ production and acted directly on the parasite. C57BL/6 female mice were infected with 1 × 10^3^ trypomastigotes of *T. cruzi* (Y strain) and treated with Zi from day 3 to day 10 after infection. On the indicated day after infection, the IFN-γ levels in serum were investigated by ELISA (A); IFN-γ mRNA expression was also investigated during infection kinetics on the heart (B); effect of Zi treatment on trypanocidal activity assay (epimastigotes) at 72 h postinfection. DMSO was utilized as the negative control (C). Parasitemia in mice infected with trypomastigotes pretreated or not with Zi for 2 h before infection (D). Parasitemia in SV129 (E) and in 5-LO knockout (F) mice. * *p* < 0.05; *** *p* < 0.001 (* representation between different groups).

Due to the decreased parasite load found at the peak of parasitemia upon Zi treatment, levels of IFN-γ, a key cytokine for the control of parasite replication/death, were evaluated systemically and in the heart. Increased levels of IFN-γ were observed in Zi-treated infected mice only at 15 dpi in serum when compared with untreated infected mice (Figure 2A). Notably, Zi treatment did not increase the expression of IFN-γ in the heart during the evaluated infection kinetics (Figure 2B). This suggests that other mechanisms of immune response and/or direct effects on the parasite are responsible for better control of the parasite load after Zi treatment in parasitemia in the initial phase of infection. To test this hypothesis, the effect of Zi was tested *in vitro* upon parasites, incubating the parasites with medium alone, Zi, or dimethyl sulfoxide (DMSO, employed as a control since it was used to dilute Zi). Zi reduced the number of parasites in the culture compared to *T. cruzi* treated with the medium or DMSO in a dose-dependent manner, with an IC_50_ value of 231.2 mM (Figure 2C). Mice infected with trypomastigotes pretreated with Zi demonstrated reduced parasitemia compared with those of mice infected with untreated trypomastigotes (Figure 2D). Notably, we tested the effect of Zi on epimastigotes of the CL-Brener strain and obtained a similar phenotype (Figure S1). To test the effect of Zi on parasites *in vivo*, excluding its effect on the host 5-LO enzyme, 5-LO knockout mice were infected and treated with Zi during the afferent phase of infection. As shown in Figure 2E, infected WT mice (Sv129 background) treated with Zi had a reduced number of trypomastigotes at 9 dpi. In the absence of 5-LO, Zi also reduced the number of parasites at the peak of parasitemia (Figure 2F).

### Epoxide Hydrolase is a Potential Target Behind the Trypanocidal Effect of Zi

The unexpected trypanocidal effects of Zi led us to investigate its potential mechanisms of action to better understand its biological effects. Inverse virtual screening (IVS) analysis was performed to identify the molecular targets. The results of the ligand-based IVS suggested ten potential biological targets for Zi, including 5-LO. Among them, the enzyme epoxide hydrolase (EH) ranked second behind 5-LO. EH stands out for its diverse reported biological effects and the number of potent inhibitors of this target that have high two-dimensional (2D) or three-dimensional (3D) similarity to zileuton (Table 1). EHs play important roles in the arachidonic acid (AA) cascade via the cytochrome P450 (CYP450) pathway. The epoxygenase CYP enzymes generate four isomers of epoxyeicosatrienoic acid (EETs) by catalyzing the epoxidation of AA olefin bonds. EETs are endothelium-derived hyperpolarizing factors with several pharmacological effects.^33^ These endogenous lipid mediators are broken down into diols by soluble epoxide hydrolases, which reduce their beneficial effects. Consequently, the inhibition of EH is targeted for the discovery and development of novel anti-inflammatory drugs.^34^ EH is found in all organisms, including *T. cruzi* epimastigotes, making it an interesting target for the discovery of drugs for the treatment of other pathologies, such as Chagas disease.^35^

**Table 1:** Top Ten Results of Potential Molecular Targets for Zileuton Identified by SwissTargetPrediction

molecular target	uniprot ID^a^	CHEMBL ID^b^	similar molecules 2D/3D^c^
arachidonate 5-lipoxygenase	P09917	Chembl215	147:4
epoxide hydrolase	P34913	Chembl2409	18:1
leukotriene B4 receptor	Q15722	Chembl3911	4:1
5-lipoxygenase activating protein	P20292	Chembl4550	14:1
serine/threonine protein kinase	075460	Chembl163101	83:0
histone deacetylase 5	Q9UQL6	Chembl2563	22:0
histone deacetylase 7	Q8WUI4	Chembl2716	29:0
alkaline phosphatase placental-like	P10696	Chembl3402	6:0
phospholipase A2	Q9Y263	Chembl6114	12:0
testis-specific androgen-binding protein	P04278	Chembl3305	5:0

a Uniprot ID of the molecular targets;

b Chembl ID of the molecular targets.

c Number of molecules that are known to bind the target listed and are similar to Zileuton considering 2D/3D similarity.

To further investigate the potential interaction between Zi and EH, we performed molecular docking simulations. To obtain the 3D model of the EH, we carried out an extensive search of the literature using the TriTrypDB, BLAST, and Uniprot databases to obtain the primary sequence of *T. cruzi* EH. For this search, we used the rat liver EH sequence as the input sequence because of its similarity to the *T. cruzi* enzyme.^36^ The tertiary structure of *T. cruzi* hydrolase (gene: Tc00.1047053503399.20; UniProt ID: Q4CQ95) was built using the AlphaFold2 program with excellent model confidence, as indicated by high pLDDT values, and good geometric quality according to evaluation with MolProbity^37^ (Figure S2 and Table S1). Therefore, we used the AlphaFold 2 model to conduct molecular docking studies with DockThor.^38^

**Figure 3:**
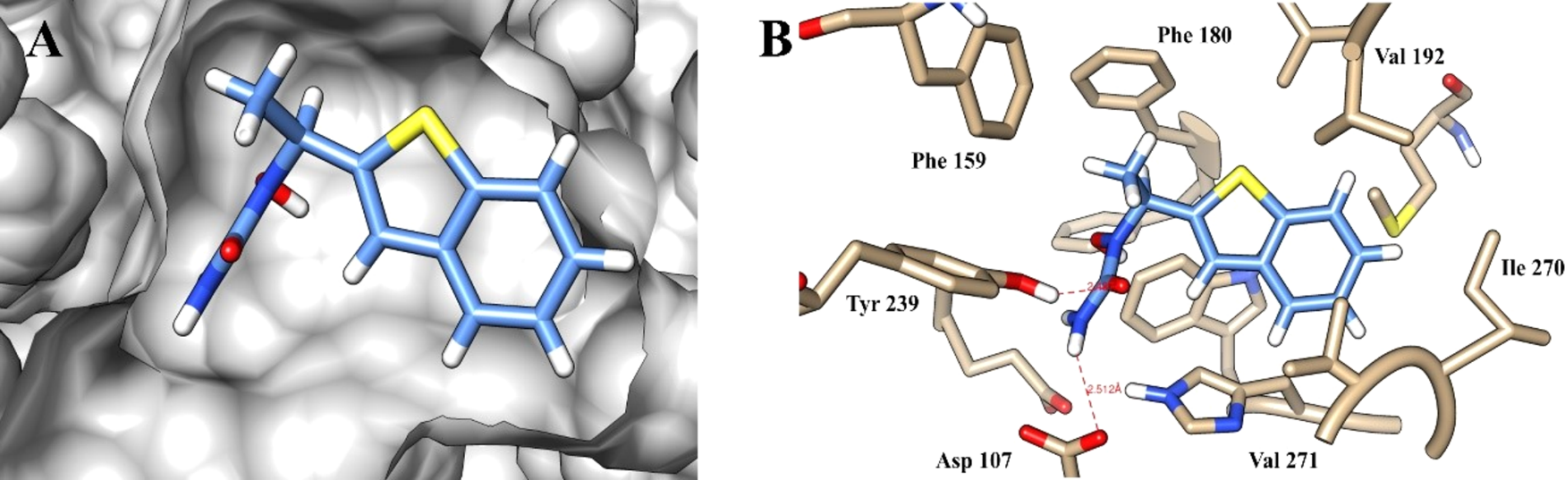
Potential binding mode of Zileuton in the *T. cruzi* epoxide hydrolase active site (A) and intermolecular interactions between the compound and amino acid residues of EH (B). Hydrogen bonds are represented by red dashed lines.

To validate our molecular docking protocol, we performed a blind redocking analysis using a crystal of human EH cocrystallized with a Zileuton analogue (PDB ID: 6YL4). We observed a very similar binding mode position between the cocrystallized ligand and redocked molecule (root-mean-square deviation (RMSD) = 1.32), validating the protocol used in this study (Figure S3). The predicted binding mode for Zi revealed three regions of the *T. cruzi* EH active site that showed good complementarity (Figure 3A). The aromatic ring of the hydrophobic drug interacted with the amino acid residues Val261 and Met 42, while the methyl group made hydrophobic interactions with Phe173, which is part of the second hydrophobic pocket. The iron-chelating moiety of Zi can form hydrogen bonds with amino acid residues Ser44 and His284 (Figure 3B).

To further analyze the proposed binding mode for Zi, we considered complexes between human EH and various Zi analogs. The docked pose of Zi resembled the experimentally observed binding modes of complexes with EH (Figure S4A,B). Soluble EH had three important binding pockets (Figure S4B). Specifically, the hydrogen bonds formed between the ligands and Asp 107, which is part of the EH hydrophilic pocket, are considered necessary for enzyme inhibition. Thus, the presence of a polar moiety is considered the central pharmacophore of EH inhibitors.^33^ Considering the molecular structure of Zi, it is expected that the iron-chelating portion interacts with the hydrophilic pocket of EH. However, the overlap between the docked pose of Zi and one of its crystallographic analogues (PDB ID: 6YL4) revealed different orientations for this polar moiety (Figure S4A). The active sites of human and *T. cruzi* EH are marked by significant differences. Polar amino acid residues such as Pro, Gln, and Asp were replaced with hydrophobic residues such as Val, Phe, and Leu in *T. cruzi* (Figure S5). These substitutions alter the polarity of the described binding pockets of human EH and, therefore, explain the different orientations of the iron-chelating moieties.

### Zi Treatment at the Intermediate Phase of *T. cruzi* Infection Reduced Innate Cells Producing IL-12 and T Lymphocytes Producing IFN-γ and IL-17 in the Spleen

**Figure 4:**
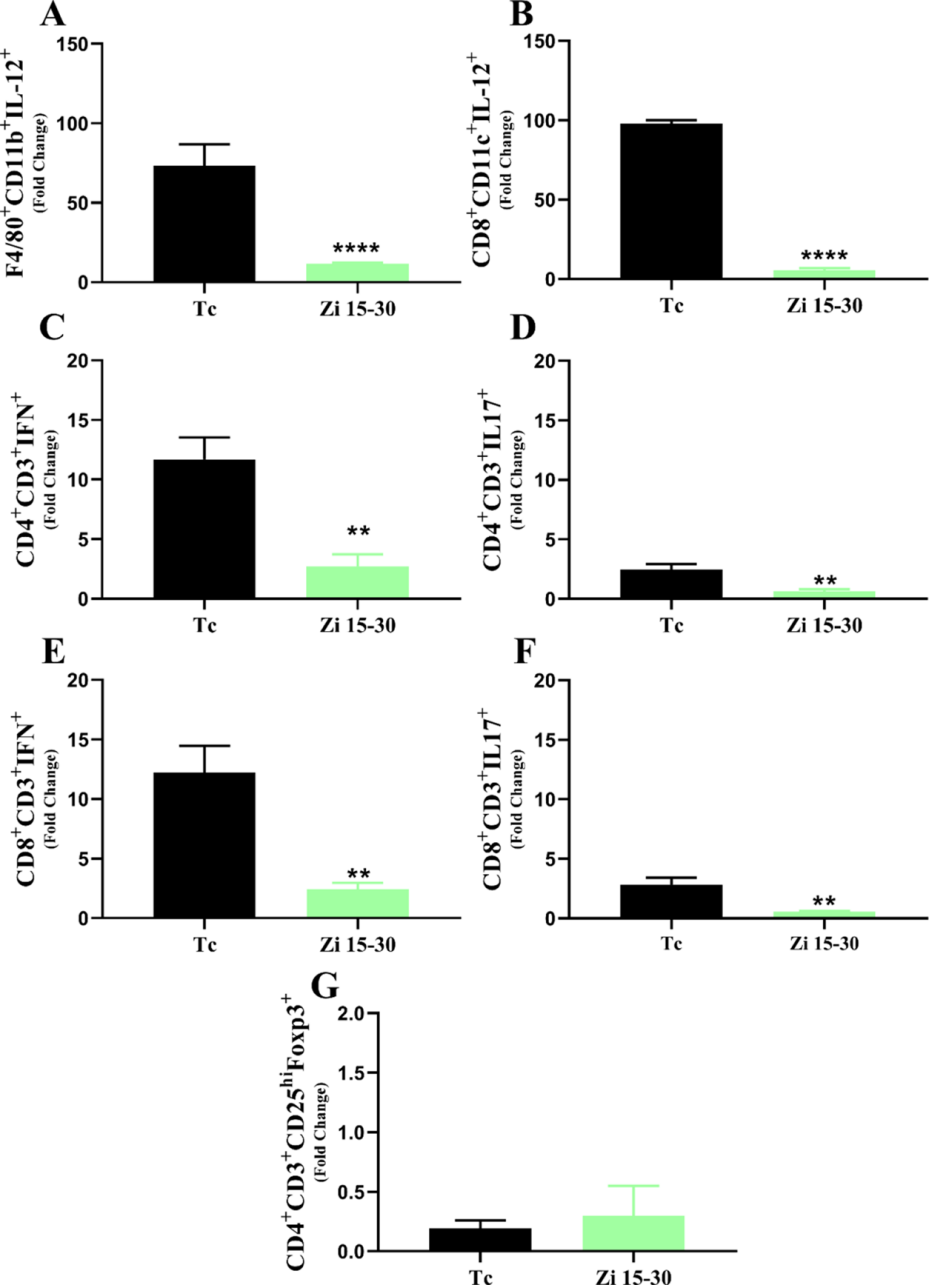
Characterization of the cell profile by flow cytometry in the spleen throughout infection. C57BL/6 female mice were infected with 1 × 10^3^ trypomastigotes of *T. cruzi* (Y strain) and treated with or without zileuton from days 15 to 30 after infection (Zi 15–30) or not (Tc). Flow cytometry analyses showing the frequency of cells in the spleen at 30 days postinfection among groups of macrophages (CD11b^+^ F480^+^) producing IL-12 (A); dendritic cells (CD8^+^CD11c^+^) producing IL-12 (B), lymphocytes (CD4^+^CD3^+^) producing IFN-γ (C); lymphocytes (CD8^+^CD3^+^) producing IFN-γ (D); lymphocytes (CD8^+^CD3^+^) producing IL-17 (E); lymphocytes (CD4^+^CD3^+^) producing IL-17 (F); and T regulatory cells (CD4^+^CD3^+^CD25hi^+^Foxp3^+^) (**G**). ** *p* < 0.01; **** *p* < 0.0001 (* representation between different groups).

In addition to unveiling the potential target underlying the trypanocidal effect of Zi, it was also important to study the time course of the anti-inflammatory properties of Zi in the *T. cruzi* infection model used in our study. To investigate the immunomodulatory effects on the organs, we analyzed the effect of Zi treatment from days 15 to 30 after infection. This treatment significantly reduced the frequency of both innate and adaptive immune responses, including macrophages and dendritic cells producing IL-12 (Figure 4A,B) and T CD4^+^ and T CD8^+^ producing IFN-γ and IL-17 (Figure 4C–F). No differences in T CD4^+^ regulatory cells were observed (Figure 4G).

### Zi Treatment at an Intermediate Phase of *T. cruzi* Infection Increased SOCS2 Expression, Decreased Inflammation, and Improves Cardiac Function

**Figure 5:**
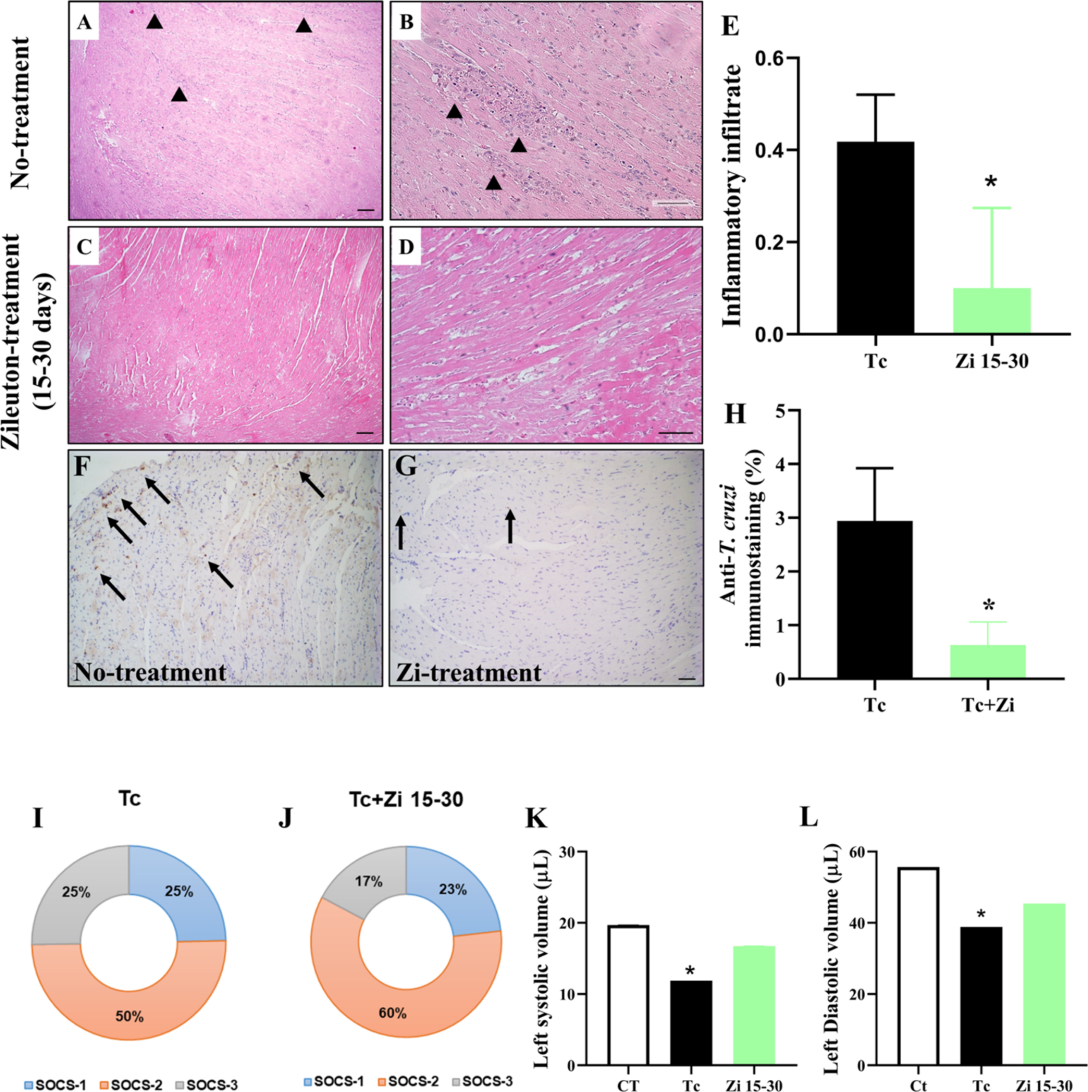
Myocardial pathology examined in Zi-treated and untreated infected mice from 15 to 30 dpi. Mice were infected with 1 × 10^3^ trypomastigotes and treated with Zi from 15 to 30 dpi. At 45 dpi, cardiac tissue sections from the untreated (A, B) and treated (C, D) mice were fixed and stained with H&E. The arrowheads indicate inflammatory tissue infiltration. The inflammatory scores of (E) are also shown. Myocardial inflammation was reduced in Zi-treated mice compared to untreated mice. Immunolabeling of paraffin-embedded cardiac tissues from infected hearts using anti-*T. cruzi* antibody (F, G) with quantification shown in (H). Arrows indicate immunostaining for anti-*T. cruzi* in cardiac tissue from mice treated with Zi (Zi treatment; 15–30 days) or untreated mice (No-treatment). Scale Bar: 3 μm. 4× and 10× objective. Analyzes of mRNA expression of SOCS1, SOCS2, and SOCS3 (I, J). The ventricular volume was measured using echocardiography (K, L). * *p* < 0.05 (* different groups).

Pathological damage to the hearts of infected mice was examined using hematoxylin and eosin (H&E) staining. H&E-stained tissue sections are the cornerstone of anatomical pathological diagnosis. In the untreated infected animals, foci of inflammation were scattered throughout the heart muscle fibers (Figure 5A,B). In contrast, treatment with Zi from days 15 to 30 dpi decreased cell infiltration (Figure 5C,D), as quantified by the histopathological score (Figure 5E). Moreover, treatment with Zi reduced the parasite load in the heart tissue (Figure 5F–H). SOCS plays an important role in the regulation of various cytokine/growth hormone-mediated processes.^39−41^ Our group has previously demonstrated that SOCS2 plays a role in *T. cruzi* infection and is crucial for controlling inflammation and heart function.^18,22^ Zi treatment significantly increased the expression of SOCS2, but not SOCS1 or SOCS3, in the heart (Figure 5I,J). Echocardiogram analysis showed that the Zi-treated animals had a heart pattern (systolic and diastolic volumes) more similar to that of the control animals than those of the untreated infected mice (Figure 5K,L).

### At the Chronic Phase of *T. cruzi* Infection, Zi Treatment Increases the Expression of SOCS2, Reduces the Heart Inflammatory Infiltrate, and Improves Cardiac Function

**Figure 6:**
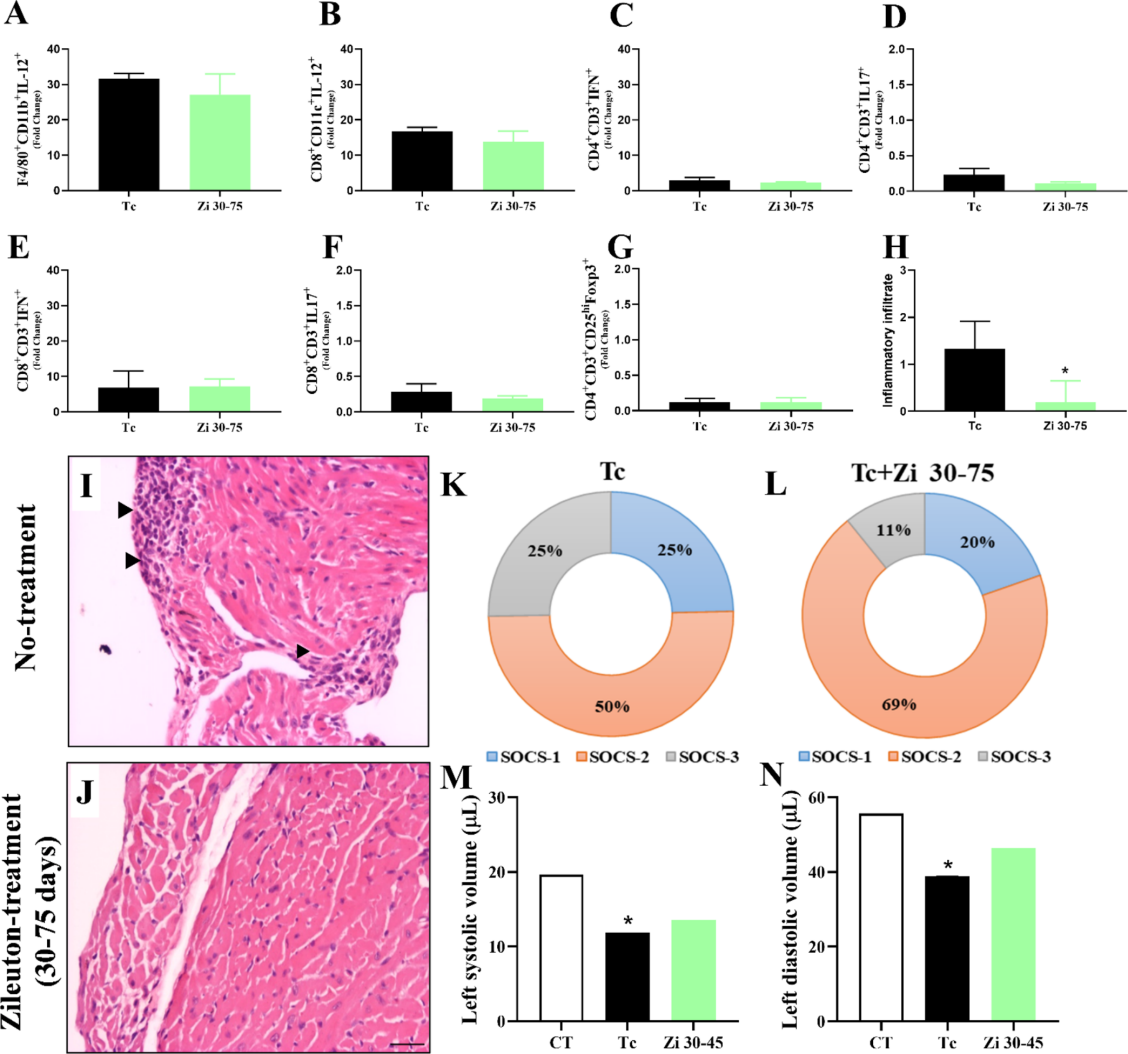
Effect of Zi treatment on heart inflammation during the *T. cruzi* chronic infection. C57BL/6 female mice were infected with 1 × 10^3^ trypomastigotes of *T. cruzi* (Y strain) and treated with Zi from days 30 to 75 after infection (Zi 30–75). Flow cytometry analyses showing the frequency of cells in the spleen at 75 days postinfection (dpi) among groups of macrophages (CD11b^+^ F480^+^) producing IL-12 (A); dendritic cells (CD8^+^CD11c^+^) producing IL-12 (B), lymphocytes (CD4^+^CD3^+^) producing IFN-γ (C); lymphocytes (CD8^+^CD3^+^) producing IFN-γ (D); lymphocytes (CD8^+^CD3^+^) producing IL-17 (E); lymphocytes (CD4^+^CD3^+^) producing IL-17 (F); and T regulatory cells (CD4^+^CD3^+^CD25^hi^Foxp3^+^) (G). Myocardial pathology was examined in untreated and Zi-treated infected mice at 30–75 dpi. At 75 dpi, sections of cardiac tissue from untreated (H) and Zi-treated 30–75 dpi (I) mice were fixed and stained with H&E. The arrowheads indicate inflammatory tissue infiltration. The inflammatory scores for (J) are also shown. Myocardial inflammation was reduced in Zi-treated mice compared to untreated mice. Scale Bar: 3 μm. 10× objective. Analyzes of mRNA expression of SOCS1, SOCS2, and SOCS3 (K, L). The ventricular volume was measured using echocardiography at 45 dpi (M, N). * *p* < 0.05 (* different groups).

The main problem associated with Chagas disease is cardiomyopathy, and treatment with Bz and nifurtimox does not affect the development of cardiac disease in the chronic phase.^42,43^ Therefore, we administered Zi during the early chronic phase of the disease from day 30 to day 75 after infection. No differences were observed in the profiles of innate and adaptive immune cells (the same populations analyzed in the other treatment approaches) in the spleen when compared with the untreated infected mice (Figure 6A–G). However, histological analyses revealed that Zi treatment in the chronic phase of the disease reduced the number of inflammatory cells in the heart (Figure 6H–J). Notably, this treatment increased the expression of SOCS2, but not SOCS1 or SOCS3, in the heart compared with that in untreated infected mice (Figure 6K,L). Echocardiogram analysis showed that the Zi-treated animals had a heart pattern (systolic and diastolic volumes) more similar to that of the control animals than did the untreated mice (Figure 6M,N).

## Discussion and Conclusions

Recent studies have demonstrated the role of 5-LO in the development of heart abnormalities in experimental Chagas disease.^44,45^ To the best of our knowledge, this is the first study investigating the effects of a treatment featuring selective inhibitors of this enzyme.

Our initial results demonstrate that early treatment with Zi reduced parasitemia at its peak (9 dpi). Parasite elimination and the associated pathogenesis are closely interconnected because high parasitemia can trigger a robust immune response, which is a determinant of symptomatic Chagas disease; however, lower circulating levels of *T. cruzi* do not necessarily promote a milder disease.^46^ When we observed the immunological profile of the spleen, Zi treatment increased the percentage of Th17 cells (9 dpi), which are responsible for antimicrobial protection, maintenance of skin, intestine, and lung barrier, as well as for neutrophils recruitment, among other effects.^47^ The importance of IL-17 in *T. cruzi* infection found by Miyazaki et al. is that IL-17 knockout (KO) mice infected with *T. cruzi* presented with higher parasitemia and mortality rates than WT mice.^48^ These results corroborate our findings and suggest that one of the possible mechanisms by which Zi controls parasitemia is through the modulation of the immune response.

Among the cytokines involved in the immunological response to *T. cruzi*, IFN-γ is crucial for parasite containment, and this cytokine acts against the trypomastigotes and amastigotes forms and induces TNF and IL-1β production.^49,50^ In this regard, we verified whether treatment with Zi could stimulate IFN-γ production, thus promoting the reduction of parasitemia. Interestingly, animals treated with Zi did not show increased IFN-γ levels at 9 dpi but rather at 15 dpi when we observed no differences in parasitemia between treated and untreated groups. At this time point, we no longer observed parasites in the bloodstream, indicating that these animals began to shift toward the chronic phase of infection. In this context, we hypothesized that the circulating IFN-γ increase could contribute to a reduction in tissue parasitism since a study conducted by Canavaci and colleagues using an experimental Chagas disease model in 5-LO KO animals demonstrated that, in addition to increased survival, infected 5-LO KO mice presented lower tissue parasitism when compared with WT animals.^44^ Unlike in plasma, we observed no increase in the IFN-γ expression at any time point in the cardiac tissue, which characterizes a good prognosis because exacerbated IFN-γ production might cause tissue damage.^51^

Considering that the reduction in parasitemia was not due to IFN-γ levels, we investigated whether Zi could also act directly on the parasite. Initially, we incubated *T. cruzi* epimastigote forms in the presence of Zi, which resulted in a significant reduction in the number of parasites at 48 h post-treatment. We then incubated trypomastigote forms in the presence of Zi and infected mice with these parasites, which resulted in a reduction in parasitemia. Finally, we infected 5-LO KO animals, treated them with Zi, and observed a reduction in parasite load. Although the reduction observed in these animals was not as significant as that found in the WT mice, these results suggest that this reduction originates from the joint action of Zi, which acts directly on the parasite and modulates the immune system.

The capacity of a compound to inhibit more than one target is the basis for drug repurposing^52^ and the motivation for multitarget drug design. This approach, based on the complexity of the pathologies, considers that single-target drugs are insufficient to achieve the desired therapeutic effects, especially for complex diseases, such as those observed in Chagas disease.^53^ Recently, drug repurposing and multitarget drug design have been discussed to accelerate the discovery of safer and more effective drugs for the treatment of Chagas disease.^54^ Our results demonstrate the unexpected activity of Zi against *T. cruzi*, showing that this drug could be a promising candidate with multitarget properties for the treatment of this pathology.

Several studies have recognized the importance of EH, including the synthesis of analogues using a hybridization strategy that incorporates the iron-chelating moiety of Zi and urea. Hybrid molecules have been described as dual EH/5-LO inhibitors and have been discussed not only as effective anti-inflammatory compounds but also as safer antihypertensive prototypes.^55^ The existence of EH inhibitors that contain part of the chemical structure of Zi justifies the SwissTargetPrediction results and reinforces the idea that EH could be another target for this drug.

EHs perform different functions depending on their localization site and^35^ participate in three main biological roles: detoxification, catabolism, and signaling molecule regulations. The involvement of EHs in these crucial cellular functions has motivated interest in the use of EH inhibitors to treat infectious diseases. The importance of EH in the survival of *T. cruzi* epimastigotes has also been described. This enzyme is crucial for drug detoxification by *T. cruzi* epimastigotes. The compilation of *in silico* results and data from the literature^37^ provides strong evidence that the inhibition of EH by Zi may be the potential molecular pathway underlying the trypanocidal effect demonstrated by *in vitro* assays.

CCC is the most important clinical manifestation of *T. cruzi* infection in humans owing to its severity, morbidity, and mortality.^46^ Therefore, the study of cardiac tissues is important for investigating new treatments for this disease. Proposing a treatment for Chagas disease involves the challenge of demonstrating that it is effective in different phases of the disease, given that as it is a neglected disease, its diagnosis often occurs at a late stage. We observed that Zi treatment at intervals of 15–30 dpi promoted major changes in the inflammatory cellular profile of the spleen, with a reduction in innate cells producing IL-12 and T lymphocytes producing IFN-γ and IL-17. In *T. cruzi* infection, the cytokine IL-12 is necessary for the differentiation and clonal expansion of Th1, T CD8^+^, and B cells.^50^ Therefore, the reduction of cytokines observed in the Zi treatment is important because, although they are of great importance to the parasite, they can cause tissue damage in large quantities.^56,57^ Concomitant to the reduction in cells producing IL-12, IFN-γ, and IL-17, we observed that Zi treatment induced an improvement in the inflammatory process of cardiac tissue and promoted cardiac function more similar to that of uninfected mice. Notably, our group demonstrated that treatment with Zi in animals infected with murine *Betacoronavirus* also triggered an improvement in cardiac function, maintaining a profile closer to that of uninfected animals.^29^ Furthermore, a study demonstrated that Zi protected mice from cardiac hypertrophy induced by pressure overload with concomitant fibrosis in a time- and dose-dependent manner through a mechanism dependent on excessive oxidative stress.^58^ Another study using 5-LO KO mice infected with the Y strain of *T. cruzi* demonstrated that infected 5-LO KO mice exhibited lower levels of leukocytes and cytokines in the myocardium, reduced levels of myocardial fibrosis, and consequently reduced tissue rearrangement.^45^

Notably, we observed that intermediate treatment with Zi increased the level of SOCS2 expression. It is an important regulator of other members of the SOCS family, such as SOCS1 and SOCS3.^19,59^ It also regulates the Janus kinase-dependent signaling pathway and cytokine (JAK)/signal transducers and activators of transcription (STAT) in cells of the immune system, and modulate the production of mediators such as TNF and IL-12.^17,19,60^ Furthermore, studies from our group have demonstrated that in the acute phase of infection triggered by *T. cruzi*, SOCS2 deficiency promotes a general anti-inflammatory phenotype, which is accompanied by changes in L-type Ca^2+^ and K^+^ currents in cardiomyocytes, that cause significant cardiac impairment.^18^ Furthermore, we observed that SOCS2 is important for the expansion and generation of different cellular profiles and interferes with lymphocyte apoptosis and the inflammatory response to *T. cruzi* in both hematopoietic and nonhematopoietic cells.^22^ Together, these data reinforce the importance of inducing SOCS2 through Zi, demonstrating that one of the possible mechanisms by which Zi improves cardiac function and attenuates tissue damage is by inducing SOCS2. Although treatment in the chronic phase at 30–75 dpi did not promote significant changes in the immune cellular profiles studied here, the treatment maintained a reduction in the inflammatory score in cardiac tissue, was associated with improved cardiac function, and induced an increase in SOCS2 expression, which corroborates the hypothesis that the improvement in cardiac function and tissue organization may be due to the induction of this protein.

## Conclusions

Our findings show that treatment with Zi is a promising tool for the therapeutic management of Chagas disease as it interferes with parasite replication, modulates local and systemic immune responses, and is a beneficial treatment option compared to existing drugs. Additionally, it can potentially be used as an adjuvant in combination with the currently used drugs.

## Materials and Methods

### Mice

Female C57BL/6 Wild-type (WT) mice aged 6–8 weeks were obtained from Centro de Bioterismo (CEBIO) of the Federal University of Minas Gerais (UFMG). 5-LO KO and Sv129 WT mice were obtained and maintained at the animal facility of the Department of Biochemistry and Immunology at the Institute of Biomedical Sciences (UFMG). They were housed in individually ventilated cages in an animal facility at 24 ± 2 °C with a 12/12-h light/dark cycle and were provided *ad libitum* access to water and food. All of the experimental procedures were performed at the NB2 facility. This study was conducted in strict accordance with the Brazilian guidelines for animal experimentation and the recommendations of the Guide for the Care and Use of Laboratory Animals of the National Institutes of Health (NIH). The Animal Ethics Committee (CETEA) of UFMG approved all experiments and procedures (protocols 305/2016 and 15/2023).

### Parasites and Experimental Infection

The Y strain of *T. cruzi* was maintained in Swiss mice, and 1 × 10^3^ trypomastigote forms were used to infect WT mice via the intraperitoneal route. Parasitemia was determined daily in 5 μL of blood collected from the tail vein. For *in vitro* stimulation studies with strain Y, trypomastigotes were grown and purified from a monkey kidney epithelial cell line (LLC-MK2). Epimastigotes from axenic cultures of the CL-Brener strain were grown in a liver infusion tryptose (LIT) medium and used for *in vitro* experiments.

### Drug Treatments

Zileuton was obtained from ZyFlo Critical Therapeutics, Inc. (Lexington, Massachusetts). Zileuton was dissolved in 0.5% sodium carboxymethyl cellulose (Synth-Labsynth, Diadema, Brazil). Zileuton was orally administered at a dose of 30 mg/kg (once a day). Treatments were performed during *T. cruzi* infection and after infection at specific time points (3–10, 15–30, and 30–75 days).

### Serum Cytokine Assays

Mice blood samples were collected at several time points postinfection and were centrifuged at 2000*g* for 10 min at 4 °C. In the obtained serum, IFN-γ levels were measured by enzyme-linked immunosorbent assay (ELISA) in accordance with the manufacturer’s guidelines (R&D Systems, Minneapolis, MN).

### Reverse Transcriptase Polymerase Chain Reaction

Total RNA was isolated from 50 mg of heart from *T. cruzi* infected and control mice and homogenized in 500 μL of Brazol reagent (LGC Biotecnologia, Cotia, SP, Brazil) according to the manufacturer’s instructions. For reverse transcriptase polymerase chain reaction (RT-PCR), purified RNA (2 μg) was used to synthesize complementary DNA (cDNA) using the reverse transcriptase M-MLV (Promega, Madison, WI) following the manufacturer’s instructions. cDNA was amplified in a PCR using GoTaq Green Master Mix (Promega) and specific primers for SOCS1 (Forward: 5′GCATCCCTCTTAACCCGGTAC3′; Reverse: 5′AATAAGGCGCCCCCACTTA3′), SOCS2 (Forward: 5′CGCGTCTGGCGAAAGC3′; Reverse: 5′TTCTGGAGCCTCTTTTAATTTCTCTT3′), SOCS3 (Forward: 5′TTTGCGCTTTGATTTGGTTTG3′; Reverse: 5′TGGTTATTTCTTTGGCCAGCA3′) and β-actin (Forward: 5′TGGAATCCTGTGGCATCCATGAAAC3′; Reverse: 5′TAAAACGCAGCTCAGTAACAGTCCG3′). The amplification program was performed in a thermocycler PTC-100 (MJ Research, Watertown, MA), which included an initial denaturation at 95 °C for 2 min, followed by 34 cycles of 94 °C for 1 min, 60 °C for 1 min, and 72 °C for 1 min, with a final extension step at 72 °C for 2 min. The amplification products were analyzed by electrophoresis on a 2% agarose gel, visualized after staining with ethidium bromide, and quantified using ImageJ 1.48 V.

### Flow Cytometry Analysis

Splenocytes were purified from *T. cruzi*-infected mice (0, 9, 15, 30, 45, or 75 dpi) as described by Gaio et al.^22^ These cells were plated and incubated with brefeldin A (10 μg/mL) (Invitrogen). After 4 h, cells were fixed and stained with labeled antibodies against IFN-γ (fluorescein isothiocyanate-FITC), IL-12p70 (phycoerythrin-PE), F4/80 (FITC, PE-Cy7), CD4 (PE-Cy7, V421), CD8 (peridinin chlorophyll protein complex-Per-CP), CD11c (Per-CP, FITC), CD11b (V421), CD3 (PE-Cy7, APC-Cy7), IL-17 (APC), CD25 (PE-Cy7), FOXP3 (PE), and IL-10 (APC) (BD Biosciences/Pharmingen). Viable cells were analyzed by flow cytometry using a FACScan instrument (Becton Dickinson, San Jose, CA) and FlowJo software version 8.7 (Tree Star, Ashland, OR).

### Echocardiographic

To assess cardiac function, echocardiographic examinations were performed on animals anesthetized with 1.5% isoflurane (Visual Sonics, Toronto, Canada). The morphology of the heart was examined by using the M-mode configuration. Ejection fraction measurements were performed using the two-dimensional B mode according to the Simpson method. All parameters were analyzed according to American Society of Echocardiography protocols.^61^

### Histopathology and Immunohistochemistry

Hearts were obtained at 0, 9, 15, 30, 45, and 75 dpi, cut transversely, washed with phosphate-buffered saline (PBS; pH 7.2), and fixed in 4% phosphate-buffered formalin. After 24 h of fixation, the tissues were embedded in paraffin, and semiconsecutive sections were stained with H&E for inflammation assessment. Inflammation was assessed in the atrial and ventricular free walls and the interventricular septum in five fields from H&E-stained section randomly chosen at 20× objective magnification, analyzing a myocardial area of 1.5 × 10^6^ μm^2^ per field. The slides were photographed using an Olympus BX51 direct light optical microscope equipped with Image-Pro Express 4.0 software (Media Cybernetics) at a resolution of 1392 × 1040 pixels. Images were transferred via a Cool SNAP-Proof Color camcorder to a computer-attached video system using Image-Pro Express version 4.0 for Windows (Media Cybernetics). For immunohistochemical analysis, cardiac tissue slides from untreated and Zi-treated *T. cruzi*-infected mice (treatment from 15 to 30 dpi) were immunostained with an anti-*T. cruzi* antibody (1:5000) (Provided by Prof. Maria Terezinha Bahia, Universidade Federal de Ouro Preto) as previously described.^62^

### Viability and Pretreatment of *T. cruzi* Parasites

Trypomastigotes from the Y strain and epimastigotes from the CL–Brenner strain of *T. cruzi* were cultured *in vitro* with Zileuton (Zi; 50–2000 μM) at 37 °C in 5% CO_2_ and 28 °C with or without dimethyl sulfoxide (DMSO) (Sigma-Aldrich), respectively. After 48 and 72 h, parasites in the supernatant were stained with erythrosine^63^ and counted using a Neubauer chamber. For the *T. cruzi* pretreatment experiment, 10^3^ trypomastigote forms were obtained from Swiss mice, as described above, and incubated in the presence or absence of Zi (800 μM) for 2 h, followed by washing with phosphate-buffered saline (1×) and used to infect C57BL/6 mice.

### Ligand-Based Target Prediction

The Zi structure in SMILES format was obtained from the PubChem database and submitted to the SwissTargetPrediction web tool (http://www.swisstargetprediction.ch/).^64^ This approach used a library of bioactive compounds from CHEMBL23, including 376,342 compounds with 580,496 related pharmacological activities.^64^ Using this large library, SwissTargetPrediction performed double-similarity quantification between the tested molecules and bioactive compounds. The 2D measurement was performed using the Tanimoto index between path-based binary fingerprints (FP2), whereas the 3D determination was based on the Manhattan distance similarity between electroshaped 5D float vectors (ES5D). Based on the number of similar 2D/3D compounds and the potency of similar molecules, the most promising targets were ranked.^64^ The top ten molecular targets were considered for further investigation.

### Molecular Docking

The 3D structure of Zileuton was drawn by using MarvinSketch 16.9.5 software (ChemAxon Ltd.). Geometric optimization was performed in the MOPAC2016 software using the semiempirical method PM7. A pH of 7.4 was considered for defining the protonation state. The three-dimensional structure of *T. cruzi* EH was obtained through comparative modeling using the AlphaFold 2 program.^37,65^ The predicted model was validated by geometric analysis using the MolProbity online server.^66^ Charges and hydrogens were added to the modeled protein using the PropKa web tool, also considering a pH of 7.4.^67^ Docking simulations were performed using the DockThor program, which employs a genetic algorithm^68^ without the addition of cofactors. Hydrogen atoms were automatically added to the ligand, and a grid large enough to encompass all amino acid residues in the EH active site was constructed (center: 9.37, 6.69, and −2.99; size: 19.60, 20.54, and 20.30; discretization: 0.25). The Zi docking simulation was performed in the standard mode, considering 1,000,000 evaluations, a population size of 750, and 24 runs. DockThor was also used to perform a blind redocking analysis. The redocked complex was then compared with the reference cocrystallized complex (PDB: 6YL4),^69^ and the root-mean-square deviation (RMSD) was calculated using the DockThor program.^68^

### Statistical Analysis

The Shapiro–Wilk test revealed that the parameters evaluated did not show a significant departure from the normal distribution. The statistical significance of the differences in values between the control and treated/infected groups was assessed using Student’s *t*-test and two-way analysis of variance (ANOVA) with the Bonferroni post-test. Differences were considered statistically significant at *p* ≤ 0.05. GraphPad Prism software (version 8.0) was used for statistical analyses. Data are shown as the mean ± standard deviation (SEM).

## Supporting Information Available

Axenic culture of CL-Brener strain epimastigotes cultured with Zileuton (S1); predicted model of the tridimensional structure of *T. cruzi* EH made using AlphaFold 2 (S2A) and its predicted aligned error (PAE) data (S2B); protein geometry statistics calculated through Molprobity analysis (Table S1); overlap between the redocked complex and human EH (S3); comparison between the binding mode position of zileuton and the cocrystallized ligand of human EH (S4A) and the overlay of different EH inhibitors (S4B); and comparison of amino acid residues of the overlapping predicted model by AlphaFold 2 and the template crystal (S5) (PDF)
