# Supplementary material for: Zileuton, a 5-Lypoxigenase Inhibitor, is Antiparasitic and Prevents Inflammation in the Chronic Stage of Heart Chagas Disease
Source: ACS Infect Dis. 2024 Nov 28;10(12):4258–70. doi: 10.1021/acsinfecdis.4c00623 (PMC11650774; doi:10.1021/acsinfecdis.4c00623)
Supplement: Supplementary file 1 — id4c00623_si_001.pdf [file id4c00623_si_001.pdf]

Supporting Information

ZILEUTON, A 5-LYPOXIGENASE INHIBITOR, IS ANTIPARASITIC AND PREVENTS INFLAMMATION IN THE CHRONIC STAGE OF HEART CHAGAS' DISEASE

Mayra Fernanda Ricci[a#], Estela Mariana Guimarães Lourenço[a#], Rafaela das Dores Pereira[a#], Ronan Ricardo Sabino Araújo[a], Fernando Bento Rodrigues Oliveira[a], Elany Barbosa da Silva[a], Gabriel Stephani de Oliveira[b], Mauro Martins Teixeira[a,c], Nazareth de Novaes Rocha[d], Felipe Santiago Chambergo[e], Danilo Roman-Campos[f], Jader Santos Cruz[a], Rafaela Salgado Ferreira[a], and Fabiana Simão Machado[a,c*]

[a]Department of Biochemistry and Immunology, Institute of Biological Sciences, Universidade Federal de Minas Gerais, Belo Horizonte, Minas Gerais, Brazil.

[b]Department of Microbiology, Institute of Biomedical Sciences, Universidade de São Paulo, São Paulo, Brazil.

[c]Program in Health Sciences: Infectious Diseases and Tropical Medicine/Interdisciplinary Laboratory of Medical Investigation, Faculty of Medicine, Universidade Federal de Minas Gerais, Belo Horizonte, MG, Brazil.

[d]Department of Physiology and Pharmacology, Biomedical Institute, Universidade Federal Fluminense, Niterói, Rio de Janeiro, Brazil.

[e]School of Arts, Sciences and Humanities, Universidade de São Paulo, São Paulo, Brazil

[f]Department of Biophysics, Universidade Federal de São Paulo, São Paulo, Brazil.

[#]These authors contributed equally to this work.

***Corresponding author:** Fabiana Simão Machado, **e-mail:** machadofs@icb.ufmg.br

ORCID: https://orcid.org/0000-0001-9272-5209

Department of Biochemistry and Immunology

Federal University of Minas Gerais
Av. Pres. Antônio Carlos, 6627 – Pampulha, CEP 31270-901, Belo Horizonte, MG, Brazil

Supporting Figures

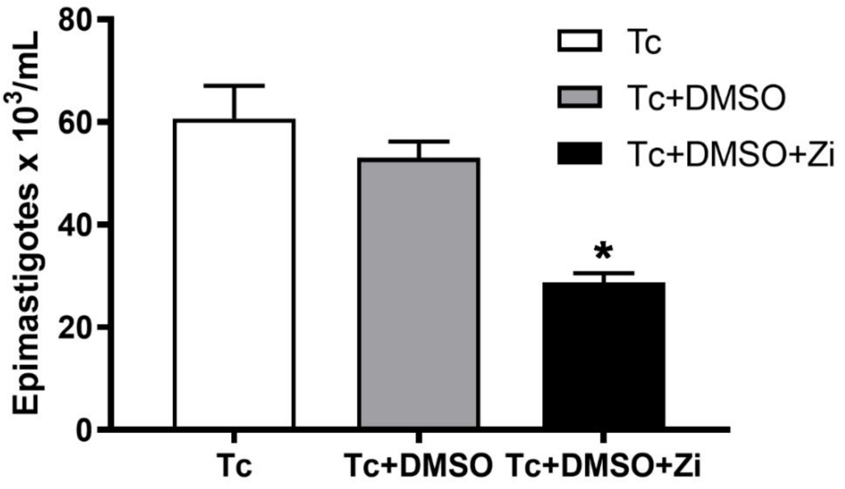

Figure S1: Axenic culture of CL-Brener strain epimastigotes Epimastigotes cultured *in vitro* with Zileuton (800 µM) diluted in Dimethyl sulfoxide (DMSO) or only with DMSO. After 48 h, parasites in the supernatant were counted. * $p < 0.05$.

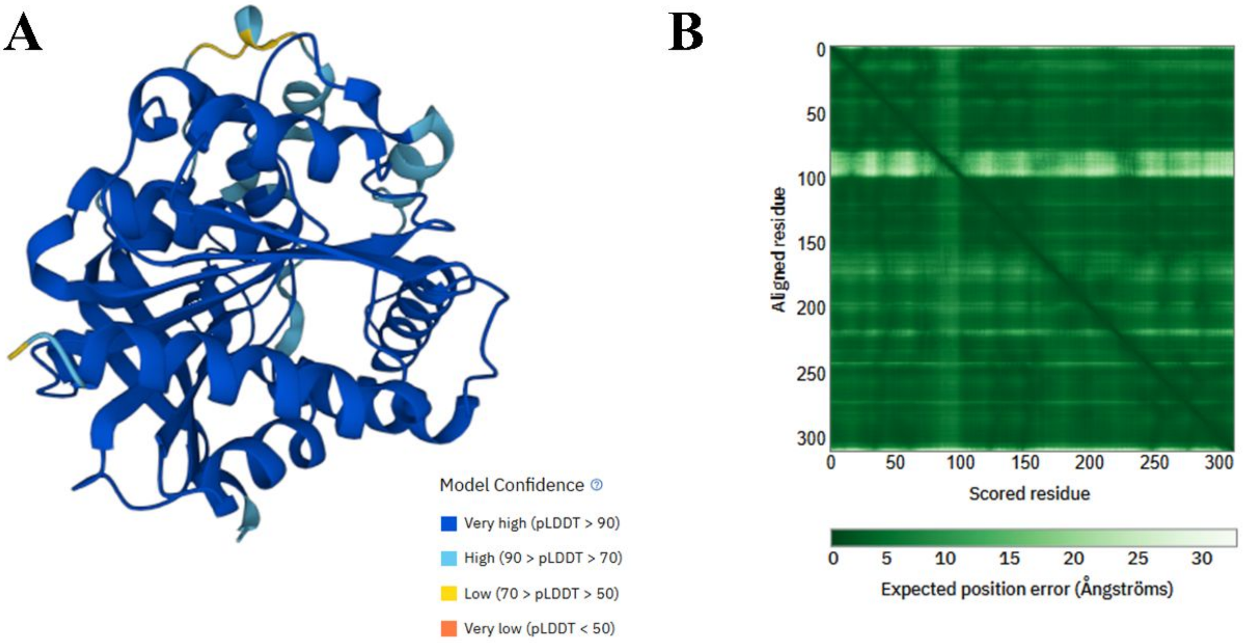

Figure S2: Predicted model of the tridimensional structure of *T. cruzi* EH made using AlphaFold2 (**A**) and its predicted aligned error (PAE) data (**B**). Colors represent pIDDT values (AlphaFold); blue indicates pIDDT > 90, and a very high prediction quality.

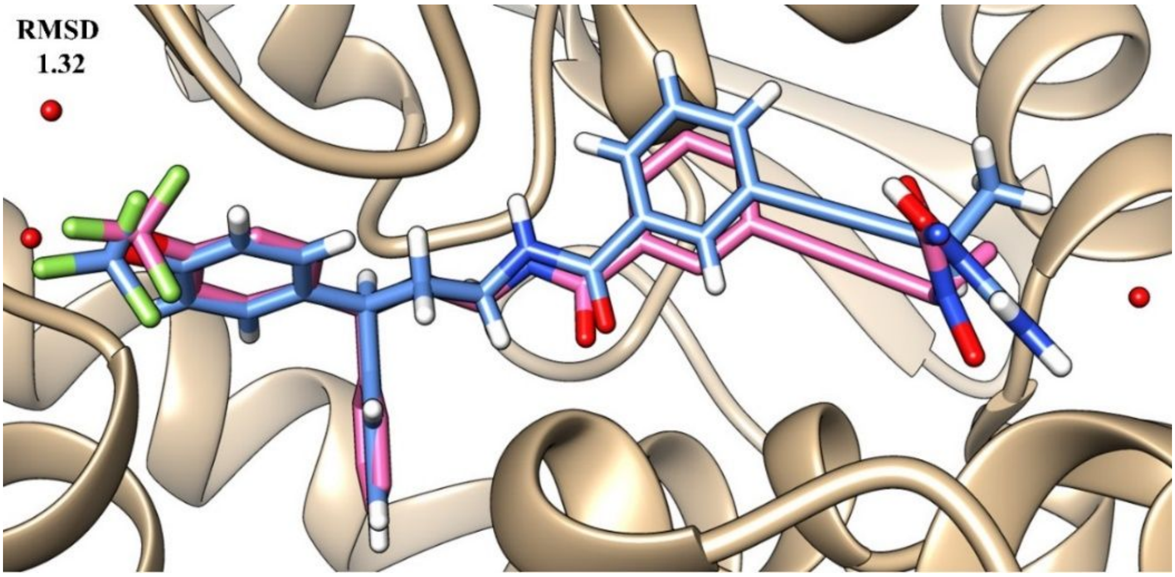

Figure S3: Overlap between the redocked complex (pink) and human EH (blue), with the RMSD resulting from this overlap.

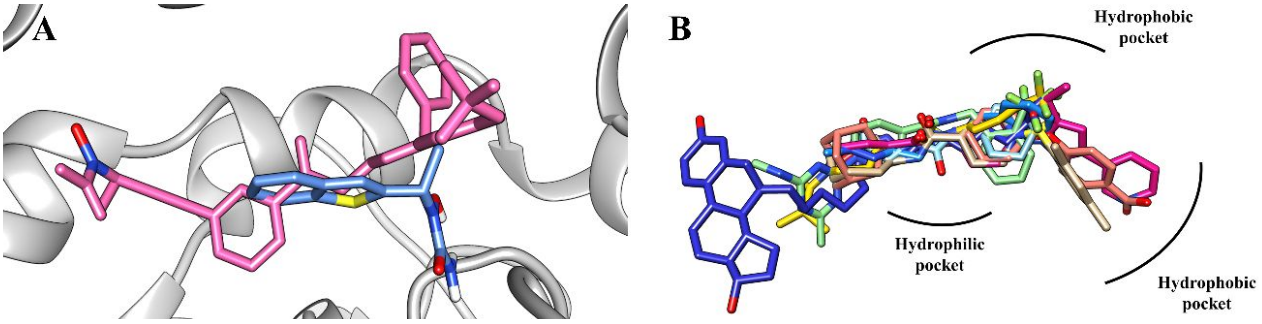

Figure S4: Comparison between the binding mode position of zileuton (blue) and the co-crystallized ligand of human EH (PDB: 6YL4, pink) (**A**) and the overlay of different EH inhibitors (**B**).

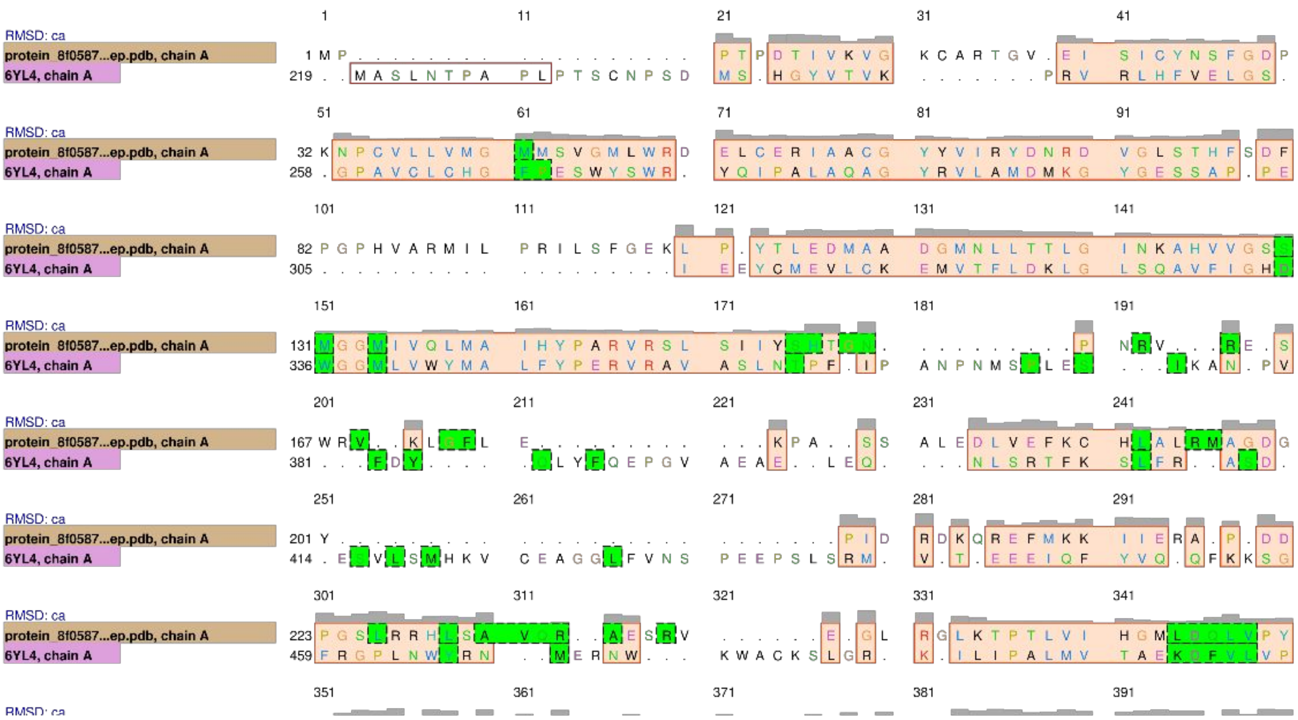

Figure S5: Comparison of amino acid residues of the overlapping predicted model by AlphaFold 2 and the template crystal. Amino acid residues of the EH active site are highlighted in green.

Supporting Table

Table S1: Protein Geometry statistics calculated through Molprobity analysis.

**Protein Geometry**	**Poor rotamers**	1	0.38%	Goal: <0.3%
	**Favored rotamers**	261	98.86%	Goal: >98%
	**Ramachandran outliers**	0	0.00%	Goal: <0.05%
	**Ramachandran favored**	302	97.42%	Goal: >98%
	**Rama distribution Z-score**	0.21 ± 0.44		Goal: abs(Z score) < 2
	**MolProbity score^**	1.03		100th percentile* (N=27675, 0Å - 99Å)
	**Cβ deviations >0.25Å**	1	0.35%	Goal: 0
	**Bad bonds:**	0/2481	0.00%	Goal: 0%
	**Bad angles:**	8/3356	0.24%	Goal: <0.1%
